# Supplementary material for: Evidence supported by Mendelian randomization: impact on inflammatory factors in knee osteoarthritis
Source: Front Med (Lausanne). 2024 May 28;11:1382836. doi: 10.3389/fmed.2024.1382836 (PMC11165061; doi:10.3389/fmed.2024.1382836)
Supplement: Supplementary file 1 [file Data_Sheet_1.ZIP › data/GCST90274835_5e6/835.leaveoneout.pdf]

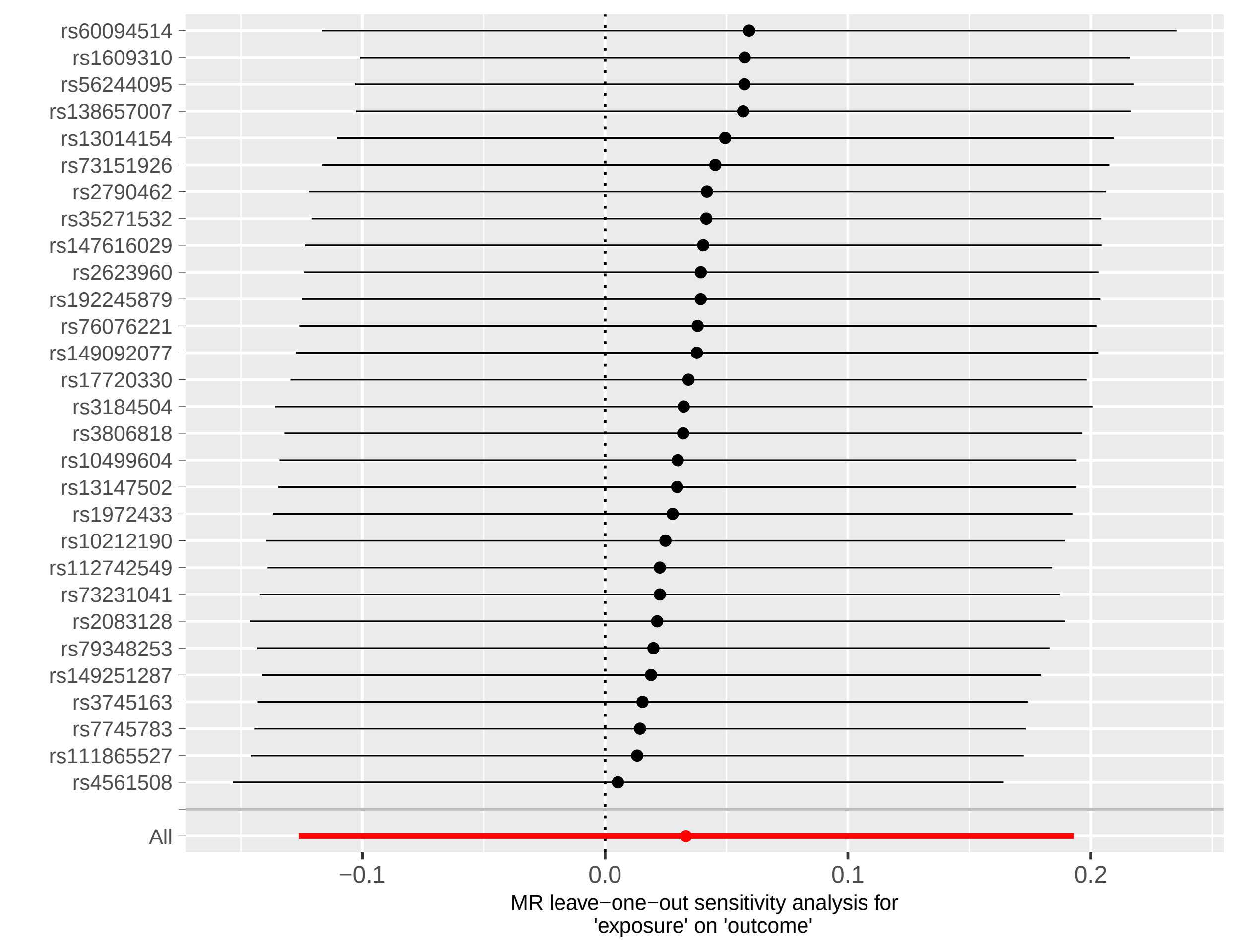
rs60094514
rs1609310
rs56244095
rs138657007
rs13014154
rs73151926
rs2790462
rs35271532
rs147616029
rs2623960
rs192245879
rs76076221
rs149092077
rs17720330
rs3184504
rs3806818
rs10499604
rs13147502
rs1972433
rs10212190
rs112742549
rs73231041
rs2083128
rs79348253
rs149251287
rs3745163
rs7745783
rs111865527
rs4561508
All
−0.1
0.0
0.1
0.2
MR leave−one−out sensitivity analysis for
'exposure' on 'outcome'
